# AALLA: Attack-Aware Logical Link Assignment Cost-Minimization Model for Protecting Software-Defined Networks against DDoS Attacks

**DOI:** 10.3390/s23218922

**Published:** 2023-11-02

**Authors:** Sameer Ali, Saw Chin Tan, Ching Kwang Lee, Zulfadzli Yusoff, Muhammad Reazul Haque, Alexios Mylonas, Nikolaos Pitropakis

**Affiliations:** 1 Faculty of Computing & Informatics (FCI), Multimedia University (MMU), Cyberjaya 63100, Malaysia; sctan1@mmu.edu.my (S.C.T.); reazul@ieee.org (M.R.H.); 2 Department of Information Technology, SZABIST University, Karachi 75600, Pakistan; 3 Faculty of Engineering (FOE), Multimedia University (MMU), Cyberjaya 63100, Malaysia; cklee@mmu.edu.my (C.K.L.); zulfadzli.yusoff@mmu.edu.my (Z.Y.); 4 School of Physics, Engineering and Computer Science (SPECS), University of Hertfordshire, Hatfield AL10 9AB, UK; a.mylonas@herts.ac.uk; 5 School of Computing, Engineering & the Build Environment, Edinburgh Napier University, Edinburgh EH10 5DT, UK; n.pitropakis@napier.ac.uk

**Keywords:** internet of things, distributed denial of service, software-defined networks, controller, ILP, AALLA

## Abstract

Software-Defined Networking (SDN), which is used in Industrial Internet of Things, uses a controller as its “network brain” located at the control plane. This uniquely distinguishes it from the traditional networking paradigms because it provides a global view of the entire network. In SDN, the controller can become a single point of failure, which may cause the whole network service to be compromised. Also, data packet transmission between controllers and switches could be impaired by natural disasters, causing hardware malfunctioning or Distributed Denial of Service (DDoS) attacks. Thus, SDN controllers are vulnerable to both hardware and software failures. To overcome this single point of failure in SDN, this paper proposes an attack-aware logical link assignment (AALLA) mathematical model with the ultimate aim of restoring the SDN network by using logical link assignment from switches to the cluster (backup) controllers. We formulate the AALLA model in integer linear programming (ILP), which restores the disrupted SDN network availability by assigning the logical links to the cluster (backup) controllers. More precisely, given a set of switches that are managed by the controller(s), this model simultaneously determines the optimal cost for controllers, links, and switches.

## 1. Introduction

Software-Defined Networking (SDN) has been attracting attention in data centre network operators, academia, and industry for its programmability and agility. SDN empowers smart industries, such as Industrial Internet of Things, with central network device configuration and administration by providing a global view of the network. The SDN framework is regarded as the hardware-less networking paradigm in which networking through programming is possible. Compared to traditional networking, in SDN technology the control and data planes are decoupled, which make it more agile in terms of networking management. A controller is responsible for the management of the entire network, whereas networking switches are responsible for operating based on the instructions deployed through controllers [1]. One of the factors for network performance and scalability is how the network is being designed [2]. The SDN architecture is flexible and can be programmed using any high-level programming language to serve the purpose of the client devices and end-users [3]. The SDN platform is not only capable of providing high performance, but also providing energy efficiency and network security [4]. However, it is necessary to counter Distributed Denial of Service (DDoS) attacks by employing controller clustering methods to control the efficiency and performance from the view of entire network security [3,4]. Due to the central location of the controller, many security concerns caused by a single point of failure have been reported [5]. Firstly, the SDN control plane is unable to handle all the flow requests due to resource consumption or malicious traffic resulting from DDoS. Secondly, the fake flow request from switches can generate several unnecessary flow rules, which makes it difficult for the data plane to store flow rules for a normal flow of traffic [6,7].

In this research study, the logical links in AALLA model are used to provide connectivity and restore the availability of resources when DDoS attacks happen. The AALLA model considers a link assignment technique and is capable of restoring the network service availability under a disruption of existing links. When a given switch is affected by a DDoS attack, the logical links will take up the switch using the backup links connecting another available port on the switch. This will restore or resume the disrupted service again to the requested users, ensuring service availability. The past literature has focused on the security of controller placement, the security of message transformation, bandwidth optimization, and network scalability [8,9,10,11,12,13,14,15,16,17,18,19]. However, the past literature has not focused on link assignment strategies considering bandwidth and cost optimization under single points of failure in SDN networks. AALLA considers metrics such as latency, throughput, cost optimization for links, switches, and controllers along with the high availability (HA) of network services in the SDN environment.

**Figure 1:**
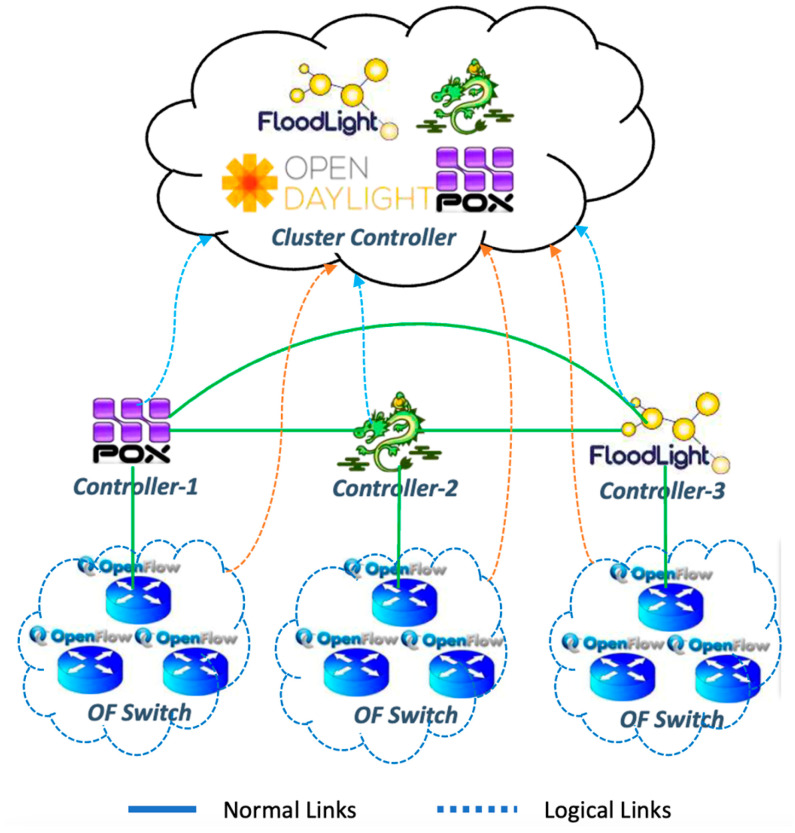
Controller cluster using logical link assignment in SDN network.

Security has been regarded as a detrimental factor in the development of SDN networks [20]. Among the security requirements of SDN networks, undisrupted availability is critical since the core function of SDN is to provide uninterrupted network services and resources. DDoS flooding attacks are the culprit in destroying availability in SDN networks [20,21]. DDoS attacks are created by two or more systems or botnets. A botnet is a compromised host system created when a computer is penetrated by software from a malware code [20]. It is essential to ensure SDN network availability for its end-users under DDoS flooding attacks. Current DDoS attacks have many forms, e.g., consumption of computational resources, disruption of configuration information, etc. [22]. To improve scalability and performance and avoid a single point of failure, the control plane is implemented as a distributed system with a cluster of controllers [23]. The hierarchy of controllers using controller clustering system is proposed as shown in Figure 1. More than one controller in SDN will serve as backup support controllers and also distribute the load of flow requests from switches.

The controller cluster is an SDN failover mechanism and proposes attack-aware logical link assignment from switches to the cluster (backup) controller under DDoS attacks. Our contributions are to formulate the logical link assignment using integer linear programming (ILP) with the intention of minimizing the cost of controllers, switches, and links. The derived model will provide a necessary tool to restore the SDN network to overcome a single point of failure.

This research paper makes the following contributions:

- We introduce the AALLA model, which aims to address the single-point-of-failure susceptibility in SDN networks exploited by DDoS attacks. The model utilizes logical link assignment from switches to backup controllers to restore the network’s availability.
- We formulate the AALLA model as an integer linear programming (ILP) problem. The model simultaneously determines the optimal cost for controllers, links, and switches while restoring the disrupted SDN network.
- Our model specifically aims to minimize the cost of controllers, links, and switches in the SDN network.

By formulating the problem as an ILP, we provide a tool that can be used to optimize the allocation of resources to the requested end-users in order to overcome the single point of failure and ensure availability of services. The rest of this paper is organized as follows. Section 2 reviews the related work on the SDN and DDoS attacks. The proposed attack-aware logical link assignment (AALLA) model is presented in Section 3 followed by the simulation results in Section 4. Conclusions are given in Section 5.

## 2. Related Work

In this section, background work on SDN, cluster controllers, and DDoS attacks and their security-related problems and possible solutions are discussed.

The authors in [24] present a heuristic approach Pareto-based Optimal Controller (POCO), a mathematical model for small- and medium-sized networks that provides operators with Pareto optimal placements with respect to different performance metrics. The authors in [25,26,27] present an integer linear model that proposes Survivor, a controller placement strategy that considers path diversity, capacity, and failover mechanisms in network design. In [28,29], the authors proposed a mathematical model for the controller placement in SDN that determines the optimal number, location, and type of controllers. The goal of the model is to minimize the cost of the network while considering different constraints. The work in [30,31,32,33,34,35] introduces mixed-integer programming formulations for the optimal placement of multi-controller switches in virtualized Open Flow-enabled SDN networks. The authors in [36,37,38,39] address the deployment of multiple controllers that work cooperatively to control a network. The authors proposed a Dynamic Controller Provisioning Problem (DCPP). The DCPP dynamically adapts the number of controllers and their locations with changing network conditions in order to minimize flow setup time and communication overhead. They formulated this problem by using integer linear programming (ILP). So far, the research studies on SDN have been focusing on the controller placement problem, scalability issues, number of controllers and reliability metrics, etc.

The authors of [40,41,42,43,44] address how the centralized paradigm of SDN is a potential vulnerability to the system assuming attackers may launch DDoS attacks against the switches and controllers. The authors further reported that an attacker may create a large number of new flows within a short period of time [21], intending to overwhelm the controller and cause network failure for legitimate users. The authors in [45,46,47] discuss how DDoS attack vulnerabilities in Open Flow SDN networks involve overpowering computing or networking resources such that a switch is unable to forward packets as expected. The authors in [48,49,50,51] illustrate controller vulnerability to flooding attacks by injecting spoofed request packets continuously; attackers deliberately generate heavy traffic to the controller, causing huge bandwidth occupation in the controller–switch channel, subsequently overloading the flow table in switches. The final goal of attackers is to downgrade or even shutdown the stability and quality of service of the network. Furthermore, the authors introduce a feasible method to protect the network against DDoS attacks more effectively. The authors in [52,53,54,55,56] investigate how an SDN can be utilized to overcome difficulties and effectively block legitimate-looking DDoS attacks mounted by a larger number of bots. Specifically, they discuss a DDoS-blocking application that runs over the SDN controller while using the standard Open Flow interface.

In the work in [50,57,58,59,60], some of the authors proposed a novel clustered distributed controller architecture in a real setting of SDN. The distributed cluster implementation comprises multiple popular SDN controllers. The proposed mechanism is evaluated using a real-world network topology running on top of an emulated SDN environment. Their proposed architecture [61,62,63] is based on distributed controller clustering in SDN that consists of two different types of controllers: an open-source and a commercial-based controllers. Both types of controllers manage different SDN networks. Each controller is set up within a cluster of three nodes; the controllers in each cluster are configured in active mode with one of the controllers acting as the primary controller. The authors of [64,65,66,67] address cluster hierarchy and highlight that the implementation of a controller cluster is outside the scope of Open Flow specifications. Their research primarily focused on providing the distributed controllers in SDN and proposed the concept of hierarchy of controllers. However, the hierarchy of controllers and attack-aware logical link assignment needs to be examined to address to the single point of failure in SDN networks. In this article, the AALLA model will provide the logical link assignment from networking devices to the cluster (backup) controllers. This mechanism of controller clustering will address the single point of failure under DDoS attacks.

A trust mechanism designed to enhance security protection in SDN-based IoT networks is proposed in [12]. The authors proposed a method to evaluate the trust level of IoT devices based on their operational behaviours and characteristics, allowing the SDN controller to actively monitor and block abnormal devices. The study in [13] presents the DARFESS (Attack-Resilient Framework for Energy Security System), which uses software-defined networking to monitor and control the cyber infrastructure of power systems. Authors provide insights into the security landscape of SDN in [14], aiming to enhance the security posture of SDN deployments and address the evolving security challenges posed by the programmability and centralization of network control. Another study in [15] presents an optimized AI model that effectively detects and mitigates DDoS attacks in SDN environments, showcasing its superiority over traditional machine learning models. The work in [16] emphasizes the significance of a dynamic controller configuration in enhancing the security and resilience of SDN networks, particularly in SCADA systems, and provides insights into the implementation and effectiveness of this approach. The focus of [17] is on the security aspects of distributed SDN controllers in an enterprise SD-WLAN. The authors highlight the security, scalability, reliability, and consistency issues associated with this design.

Currently, research in SDN has been focusing on controller placement strategies, determining the number of controllers, the location for controller placement, and scaling SDN networks. However, failover techniques using attack-aware link assignment methods aiming to mitigate DDoS-based large volumetric attacks have not been investigated thoroughly in SDN, e.g., in [8,9,10,11,12,13,14,15,16,17,18,19]. This work focuses on tackling a single point of failure and studying the hierarchy of controllers in SDN and presents a novel mathematical model for logical link assignment from switches to the cluster (backup) controllers under DDoS attacks.

So far, none of the attack-aware logical link assignment solutions proposed in the literature have taken into consideration AALLA model formulation. There are also a lot of works that study controller placement and the division of SDN networks into small domains, but not the formulation scheme that provides a solution for an attack-aware link assignment system in SDN networks under DDoS attacks. The literature review shows that the optimal controller placements only involve one or two input parameters. In the case of placing controllers using clustering, only latency is used to determine controller placement locations. Furthermore, a greedy approach that improves reliability minimizes the failure probability while keeping the shortest distance between the installed controller and switches. A framework that automatically assigns links to switches from the controllers assumes that the controllers are already placed in an SDN network.

## 3. Attack-Aware Logical Link Assignment (AALLA) Model

This section provides detailed information on the attack-aware link assignment (AALLA) model in integer linear programming (ILP) and its parameters. For the formulation, we assume that the following information is given:

1. The number of switches available in the network and the data packets (traffic) that must be sent to the controller from each switch;
2. The length and the bandwidth available for each link type to be connected between switches and controllers;
3. The characteristics of the different types of controllers. Each type of controller has a cost in USD ($), number of ports available, maximum number of requests it can handle per second, and the number of available controllers of each type;
4. The maximum link setup latency allowed for switch-to-controller communications. Based on this information, we define the following notation.

To present the notations, we define the set of switches in the network as S. These switches could be of different numbers depending on the size of the network. So, the number of switches is presented as S = {s1, s2, s3, …, sn}. Each switch can contain a number of packets in it, which are represented by $\sigma^{s}$. Each switch has a cost in USD $ and this is represented as $K^{S}$. We also define the set of controllers in the network and this is represented by C, while the number of the controllers in the set are available as C = {c1, c2, c3, …, cn}. The cost of these controllers is defined as $K^{C}$ and is represented in (cϵC) in USD $. Controllers have different numbers of ports available, represented as $\alpha^{c}$, and these are used to connect switches and other controllers. Another characteristic of the controller is that it has processing power $\mu^{c}$ for each controller in the network. There are different types of controllers, which are defined as $\partial^{C}$; some examples are Open Daylight, Floodlight, POX, etc. There are a set of links defined as L = {l1, l2, l3, …, ln} and these links have some characteristics, for example $\omega^{l}$ shows the bandwidth of the link of type (lϵL) and $\phi^{l}$ shows the price of the link of type (lϵL) in USD $.

In another scenario, we define the notations for the backup system, such as the set of backup controllers in the network as BC = {bc1, bc2, bc3, …, bcn}. The cost of the backup controller of type (bcϵBC) in USD $ is defined by $K^{bc}$ and the number of ports available in the backup controller $\left(bc\epsilon BC\right)\;are\;shown\;by\;\partial^{bc}$. Similarly the processing power of the backup controller $\left(bc\epsilon BC\right)\;is\;shown\;as\;\mu^{bc}\;\mathrm{and}$ the number of backup (cluster) controllers of type $\left(bc\epsilon BC\right)\;are\;defined\;as\;\partial^{bc}.$ Finally, the DDoS attacks are defined as ${\mathrm{DDoS}}^{s}$, which shows if the resultant number is 1 that an attack happened on a switch and if its 0 no attacks happened at all.

The proposed AALLA model has some static notations as well, which are set in the model file of the AMPL IDE setup. The maximum delay allowed in the network for flow-setup latencies is denoted by λ and the data packet size for each packet in bytes is denoted by β. “t” is the speed of light and/or communication channel. It can vary as per the medium of communication, i.e., wired or wireless. There is a function that converts the bandwidth of the link into byte/s, which is denoted by $B^{byte}$_._

The proposed AALLA mathematical model includes the following decision variables:

$$V^{l}sc=\left\{\begin{array}{cc} 1, & \;\mathrm{if}\;\mathrm{link}\;\mathrm{of}\;\mathrm{type}\;(l\epsilon L)\;\mathrm{is}\;\mathrm{installed}\;\\ & \;\;\mathrm{between}\;\mathrm{switch}\;(s\epsilon S)\;\;\\ & \;\;\;\mathrm{and}\;\mathrm{controller}\;(c\epsilon C);\;\\ 0, & \;\mathrm{otherwise}. \end{array}\right.$$

$$W^{l}cj=\left\{\begin{array}{c} 1,\;\;\;\;\;\;\;\;\;\;\;\;\mathrm{if}\;\mathrm{link}\;\mathrm{of}\;\mathrm{type}\;(l\epsilon L)\;\mathrm{is}\;\mathrm{installed}\;\\ \mathrm{between}\;\mathrm{controller}\;\left(c\epsilon C\right)\\ \mathrm{and}\;\mathrm{controller}\;\left(j\epsilon C\right);\;\\ 0,\;\;\;\;\;\;\;\;\;\;\;\;\mathrm{otherwise}. \end{array}\right.$$

$$X^{l}cbc=\left\{\begin{array}{c} 1,\;\;\;\;\;\;\;\;\;\;\;\mathrm{if}\;\mathrm{link}\;\mathrm{of}\;\mathrm{type}\;(l\epsilon L)\mathrm{is}\;\\ \mathrm{installed}\;\mathrm{between}\;\\ \mathrm{controller}\;\left(c\epsilon C\right)\;\mathrm{and}\\ \mathrm{backup}\;\mathrm{controller}\;(\mathrm{bc}\epsilon \mathrm{BC});\;\\ 0,\;\;\;\;\;\;\;\;\;\;\;\mathrm{otherwise}. \end{array}\right.$$

$$Y^{ll}sbc=\left\{\begin{array}{c} 1,\;\;\;\;\;\;\;\;\;\mathrm{if}\;\mathrm{logical}\;\mathrm{link}\;\mathrm{of}\;\mathrm{type}\;(\mathrm{ll}\epsilon \mathrm{LL})\\ \mathrm{is}\;\mathrm{installed}\;\mathrm{between}\;\\ \mathrm{switch}\;\left(s\epsilon S\right)\;\mathrm{and}\;\\ \mathrm{backup}\;\mathrm{controller}\;(\mathrm{bc}\epsilon \mathrm{BC});\;\\ 0,\;\;\;\;\;\;\;\;\;\mathrm{otherwise}. \end{array}\right.$$

$$Z^{l}sv=\left\{\begin{array}{c} 1,\;\;\;\;\;\;\;\mathrm{if}\;\mathrm{link}\;\mathrm{of}\;\mathrm{type}\;(l\epsilon L)\;\mathrm{is}\;\mathrm{installed}\\ \;\mathrm{between}\;\mathrm{switch}\;\left(s\epsilon S\right)\\ \mathrm{and}\;\mathrm{switch}\;\left(v\epsilon S\right);\;\\ 0,\;\;\;\;\;\;\;\mathrm{otherwise}. \end{array}\right.$$

### 3.1. Cost Function for AALLA Mathematical Model

The objective of the AALLA model is to optimize the cost of deploying a secure SDN network at the planning stage. This model’s development factored in the cost of controllers, switches, and links. The function space (a, b) in the following equations will calculate the distance between two points as point a and point b, where a and b refer to switches.

Equations (1) and (2) are the cost of controllers, ${\mathrm{Cost}}^{c}\left(k\right)$, and cluster (backup) controllers, ${\mathrm{Cost}}^{\mathrm{bc}}\left(k\right)$, for SDN network deployment.

(1) $${\mathrm{Cost}}^{c}\left(k\right)=\sum_{c\epsilon C}K^{C}$$

(2) $${\mathrm{Cost}}^{\mathrm{bc}}\left(k\right)=\sum_{\mathrm{bc}\epsilon \mathrm{BC}}K^{\mathrm{bc}}$$

Equation (3) is the cost of the switches, ${Cost}^{s}\left(v\right)$, connecting to the controllers.

(3) $${Cost}^{s}\left(v\right)=\sum_{s\epsilon S}K^{S}\sum_{l\epsilon L}\sum_{c\epsilon C}V^{l}sc$$

Equations (4)–(6) calculate the cost of links for connecting switches to the controller, ${Cost}^{l}\left(v\right)$; for connecting a controller to a controller, ${Cost}^{l}\left(w\right)$; and for connecting a controller to cluster (backup) controllers, ${Cost}^{l}\left(x\right)$.

(4) $${Cost}^{l}\left(v\right)=\sum_{l\epsilon L}\phi^{l}\sum_{s\epsilon S}\sum_{c\epsilon C}space\;(s,c)\;V^{l}sc$$

(5) $${Cost}^{l}\left(w\right)=\sum_{l\epsilon L}\phi^{l}\sum_{c\epsilon C}\sum_{\begin{array}{c} j\epsilon C\\ c<j \end{array}}space\;(c,j)\;W^{l}cj$$

(6) $${Cost}^{l}\left(x\right)=\sum_{l\epsilon L}\phi^{l}\sum_{c\epsilon C}\sum_{bc\epsilon BC}space\;(c,bc)\;X^{l}cbc$$

Equation (7) is the cost of links, ${Cost}^{l}\left(z\right)$, connecting switches together.

(7) $${Cost}^{l}\left(z\right)=\sum_{l\epsilon L}\phi^{l}\sum_{s\epsilon S}\sum_{\begin{array}{c} v\epsilon S\\ s<v \end{array}}space\;(s,v)\;Z^{l}sv$$

The following equation is the cost of logical links, ${Cost}^{ll}\left(y\right)$, connecting switches to the cluster (backup) controller under a DDoS attack with higher processing power of cluster (backup) controllers. This is the operating cost of the SDN network at the planning stage.

(8) $${Cost}^{ll}\left(y\right)=\sum_{ll\epsilon LL}\sum_{s\epsilon S}\sum_{bc\epsilon BC}\left(\frac{1}{\mu^{bc}}\right)Y^{ll}sbc$$

### 3.2. The AALLA Model (ILP) Formulation

Our formulation for the AALLA problem can be derived as described in this subsection. Firstly, to minimize the cost of controllers and backup controllers, the cost of links connecting switches together, the cost of switches, and the cost of linking controllers together, we devise the following Equation (9).

(9) $${(Cost}^{c}\left(k\right)+{Cost}^{bc}\left(k\right)+{Cost}^{s}\left(v\right)+{Cost}^{l}\left(v\right)+{Cost}^{l}\left(w\right)+{Cost}^{l}\left(x\right)+{Cost}^{ll}\left(y\right)+{Cost}^{l}\left(z\right))$$

This section also provides the equations that are the core part of the model to minimize the cost of SDN network deployment. Namely, the following constraint ensures that the controller has a sufficient number of ports to connect switches and other controllers.

(10) $$\sum_{c\epsilon C}\sum_{l\epsilon L}W^{l}cj+\sum_{s\epsilon S}\sum_{l\epsilon L}V^{l}sc\leq \alpha^{c}\;\;\left(j\epsilon C\right)$$

Moreover, the next constraint ensures that the link chosen between the controller and the switch can handle the bandwidth needed by the switch.

(11) $$\left(\sigma^{s},\beta \right)\sum_{l\epsilon L}V^{l}sc\leq \sum_{l\epsilon L}f\left(\omega^{l}\right)V^{l}sc\;\;\left(s\epsilon S,c\epsilon C\right)$$

Constraint (12) is obtained for the case where the round-trip flow-setup latencies for unmatched flows in each of the switches is set below or equal to λ.

(12) $$(\beta /\omega^{l}{)V}^{l}sc\leq \lambda \;\;\left(c\epsilon C,l\epsilon L,s\epsilon S\right)$$

Constraints (13) and (14) ensure all switches are connected to controllers. Constraint number (15) makes sure the switches are interconnected with their respective controller only.

(13) $$\sum_{l\epsilon L}\sum_{c\epsilon C}V^{l}sc\geq 1\;\;\left(s\epsilon S\right)$$

(14) $$\sum_{l\epsilon L}Z^{l}sv\leq 1\;\;\left(s\epsilon S,v\epsilon S,s<v\right)$$

(15) $$\sum_{l\epsilon L}V^{l}sc+\sum_{l\epsilon L}V^{l}vc\leq \sum_{l\epsilon L}Z^{l}sv+1\left(j\epsilon C,v\epsilon S,s\epsilon S\;s<v\right)$$

The constraints in Equations (16)–(18) ensure all the controllers are interconnected together, and each controller is connected to all cluster (backup) controllers using full-mesh topology (ensuring that the links from the controller to the cluster are being assigned in one direction only).

(16) $$\sum_{l\epsilon L}W^{l}cj\geq 1\;\;\left(c\epsilon C,j\epsilon C,c<j\right)$$

(17) $$\sum_{l\epsilon L}\sum_{c\epsilon C}X^{l}cbc\geq \sum_{l\epsilon L}\sum_{c\epsilon C}\sum_{\begin{array}{c} j\epsilon C\\ c<j \end{array}}W^{l}cj/2\;\;\left(k\epsilon BC\right)$$

(18) $$\sum_{l\epsilon L}X^{l}cbc\geq 1\;\;\left(j\epsilon C,k\epsilon BC\right)$$

The constraint number in Equation (19) ensures that the number of data packets that each switch sends can be processed by the controller.

(19) $$\sum_{l\epsilon L}\sum_{s\epsilon S}\sigma^{s}V^{l}sc\leq \mu^{c}\;\;\left(c\epsilon C\right)$$

The constraints in (20), (21), and (22) are for the scenario where the affected switches are connected to only one cluster (backup) controller (bcϵBC) under a DDoS attack using the logical links, and the processing power of the cluster (backup) controller is equal or at least higher than the original controller.

(20) $$\sum_{ll\epsilon LL}\sum_{j\epsilon BC}Y^{ll}sbc\leq 1\;\;\left(s\epsilon S\right)$$

(21) $$\sum_{ll\epsilon LL}\sum_{j\epsilon BC}Y^{ll}sbc\geq {\mathrm{DDoS}}^{s}\;\;\left(s\epsilon S\right)$$

(22) $$\sum_{l\epsilon L}\sum_{ll\epsilon LL}\sum_{c\epsilon C}\sum_{s\epsilon S}V^{l}sc*Y^{ll}sbc*\mu^{c}\leq \mu^{bc}\;\;\left(bc\epsilon BC\right)$$

## 4. Experimental Results and Discussion

In this section, we discuss the experimental results and simulation platform tools used for AALLA mathematical model formulation in detail.

A mathematical programming language (AMPL) was used to formulate the AALLA model along with the IBM ILOG CPLEX 12.7.0.0: optimal integer solution; this is a powerful solver for AMPL code execution. In our experiments, we used an Acer Aspire XC-780 workstation, Intel^®^ Core™ i7-6700 x64-based 6th-generation CPU @ 3.40GHz, with a memory of 8 GB RAM and virtual memory of 128 GB on Windows 10 x64.

**Figure 2:**
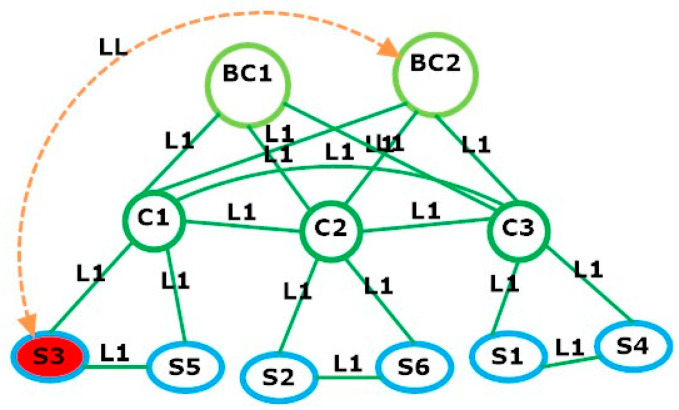
Logical link assignment between switch S3 and cluster controller BC2 under DDoS attack. Red nodes are used for nodes under attack and orange links are used to denote that a compromised switch has been restored using the next available logical link.

Table 1a,b present the two scenarios, known as problem (A) and problem (B), used for the AALLA model simulation. They consist of the following elements as depicted in Figure 1 and Figure 2.

Three controllers for problems (A) and (B) are given as C1, C2, and C3 with different specifications and a cost in USD.

Two cluster (backup) controllers BC1 and BC2 for problem (A) and problem (B) are used and they will be activated upon DDoS attack occurred on the switches connected with C1, C2, and C3.

Three type of links, L1, L2, L3, are used with different prices in USD and bandwidth in bytes, respectively. Six input switches, as S1, S2, S3, S4, S5, S6, for problem (A) and the same for problem (B) are used with a price in USD.

Some of the other constants used in this experimental setup are as follows: Beta is a constant in the model file, which is set for the size of data packets in bytes. A function of $B^{byte}$ is used as a source of converting the GBs/MBs into Bytes per second. Space/range is a function that is used to calculate the distance between two points such as point A and point B. The maximum delay is set in the model using λ, which is allowed for the flow-setup latency in the network. Delta “δ” is used for the average time in milliseconds for processing a packet in switches and “t” is used for the speed of the medium of communication, such as wired or wireless network.

**Figure 3:**
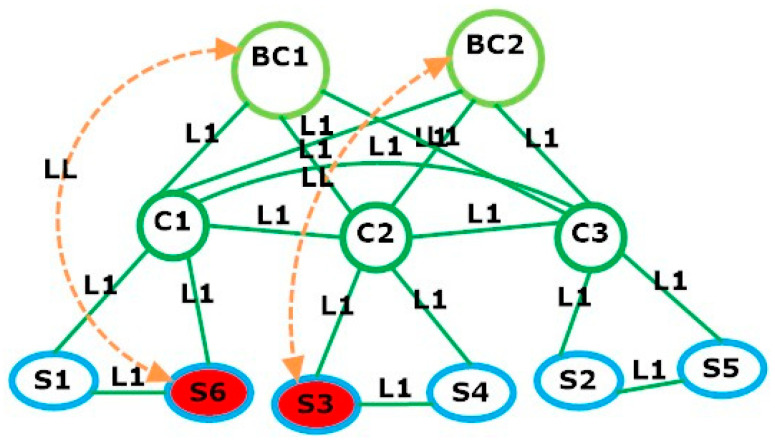
Logical link assignment from switch S3 to BC2 and from switch S6 to BC1 cluster (backup) controller under DDoS attack. Red nodes are used for nodes under attack and orange links are used to denote that a compromised switch has been restored using the next available logical link.

The simulation results are described and presented in Table 2. Here, we can observe that the total data packets are 2100 p, processed for both problems (a) and (b) with 1398 and 2986 CPLEX iterations, respectively. The DDoS attack happened on switch S3 in problem (a) in Figure 2 and switches S3 and S6 in problem (b), as illustrated in Figure 3, while a minimized cost of 45,509 USD is incurred for SDN planning.

As per the results obtained from AMPL simulation, we observed that multiple DDoS attacks in SDN networks may incur more cost in terms of restoring the networking devices to the cluster controller. The reasons are that the logical links will be chosen upon the basis of processing power; therefore, if an attack happens on a switch, then the model will choose the higher-processing-power cluster (backup) controller in order to restore the network services [68,69,70]. The results indicate that the model can be used to plan small- and medium-scale enterprises (SMEs) in SDN networks to reduce the impact of DDoS attacks and to failover a single point of failure in the SDN with optimal cost for deployment.

The experimental results provide insights into the dynamics of SDN environments, particularly in the context of mitigating DDoS attacks. One of the key findings of our study is that the occurrence of DDoS attacks within SDN networks can significantly escalate the cost associated with restoring networking devices to their respective cluster controllers. This observation highlights the critical need for proactive measures to defend against and recover from such attacks. The rationale behind the increased cost is rooted in the model’s logic, which prioritizes processing power when selecting logical links for network restoration. In the case of a DDoS attack targeting a switch, the model chooses the cluster controller with higher processing power to ensure the efficient restoration of network services. While this approach is indeed effective in terms of ensuring network resilience, it comes at an increased financial cost. This insight highlights the trade-off between network robustness and cost, a critical consideration for network administrators and decision-makers.

The findings of this research align with previous studies (e.g., [68,69,70]). The ability to model and simulate such scenarios using mathematical optimization techniques, as demonstrated in this study, provides a powerful tool for network design and operation. By using the AALLA model, small- and medium-sized enterprises (SMEs) can strategically plan their SDN networks to reduce the impact of DDoS attacks and implement cost-effective failover mechanisms.

In conclusion, our experimental results shed light on the complex interplay between DDoS attacks, network resilience, and cost considerations in SDN environments. Our model provides a tool that can be used at the planning stage of an SDN network to provide proactive defence strategies to mitigate the financial and operational consequences of DDoS attacks. The AALLA model offers a promising avenue for optimizing SDN network deployment and managing the risks associated with DDoS attacks, ultimately enhancing the overall reliability and security of modern network infrastructures.

## 5. Conclusions

In this paper, we have proposed a novel AALLA mathematical model for the attack-aware link assignment problem between the switches and cluster (backup) controllers in SDN networks. Given the set of switches in the SDN network that must be managed by the controller(s), the proposed model simultaneously determined the optimal bandwidth for the links, the assignment of the logical links to the cluster (backup) controllers under DDoS attack, as well as the interconnections between all the network elements to minimize the SDN deployment cost at the planning stage. Our simulation results have shown that this linear model performed well for the SDN network under DDoS attacks to avoid a single point of failure. We tested two input datasets with multiple attacks to analyse the results. The outcome of two problem sizes have shown that the DDoS-affected switch in scenario (a) is switch S3 and in scenario (b) the switches are S3 and S6, which were assigned to the cluster (backup) controller using logical links. This method provides the SDN network with high availability, reliability, and uninterrupted services to fulfil internet service providers (ISPs) and end-user requirements. Our plans for future work include the validation of the proposed AALLA model in real-world SDN environments. This could involve collaborating with industry partners or deploying the solutions in testbeds to assess their practicality, scalability, and performance, improving the detection and mitigation techniques for DDoS attacks in SDN networks. Also, we plan to investigate and develop more advanced controller clustering methods to enhance the resilience of SDN networks against DDoS attacks. This may include extending the current model and exploring optimal strategies for load balancing, fault tolerance, and scalability in controller clusters.

## Figures and Tables

**Table 1:** The input dataset used for AALLA model.

Controllers for AALLA Model—Problem (a)				
Controllers	$\mathrm{Alpha}\_c\;(\alpha^{c})$	$\mathrm{Mu}\_c\;(\mu^{c})$	$\mathrm{Kappa}\_c\;(K^{C})$	$\mathrm{Phi}\_c\;(\partial^{C})$
C1	8	8000	7000 USD	6
C2	16	9000	8000 USD	7
C3	32	10,000	9000 USD	4
Cluster (backup) Controllers for AALLA Model				
Controllers	$\mathrm{Alpha}\_\mathrm{bc}\;(\alpha^{bc})$	$\mathrm{Mu}\_\mathrm{bc}\;(\mu^{bc})$	$\mathrm{Kappa}\_\mathrm{bc}\;(K^{bc})$	$\mathrm{Phi}\_\mathrm{bc}\;(\partial^{bC})$
BC1	16	9900	10,000 USD	6
BC2	32	11,000	10,500 USD	7
Links for AALLA Model				
Links	$\mathrm{Omega}\_l\;(\omega^{l})$	$\mathrm{Phi}\_l\;(\phi^{l})$		
L1	10,000,000	0.28 USD		
L2	200,000,000	0.34 USD		
L3	3,000,000,000	6 USD		
Switches for AALLA Model				
Switches	$\mathrm{Sigma}\_s\;(\sigma^{s})$	$\mathrm{Kappa}\_s\;(K^{S})$		
S1	100	0.10 USD		
S2	200	0.15 USD		
S3	300	0.20 USD		
S4	400	0.30 USD		
S5	500	0.40 USD		
S6	600	0.50 USD		
Other inputs to the AALLA Model				
Input type		Symbol	Data/Units	
Data packet size		β	1700 bytes	
Function for bandwidth conversion (per second)		$B^{byte}$	1/8 per second	
Distance between two points		space	200 m	
Maximum delay		λ	349 ms	
Average time		δ	0.001 ms	
Speed of communication channel (wired or wireless)		t	0.59 per second	
Traffic intensity		p	2100 total # packets	
**Controllers for AALLA Model—Problem (b)**				
Controllers	$\mathrm{Alpha}\_c\;(\alpha^{c})$	$\mathrm{Mu}\_c\;(\mu^{c})$	$\mathrm{Kappa}\_c(K^{C})$	
C1	8	8000	7000 USD	
C2	16	9000	8000 USD	
C3	32	10,000	9000 USD	
Cluster (backup) Controllers for AALLA Model				
Controllers	$\mathrm{Alpha}\_\mathrm{bc}\;(\alpha^{bc})$	$\mathrm{Mu}\_\mathrm{bc}\;(\mu^{bc})$	$\mathrm{Kappa}\_\mathrm{bc}\;(K^{bc})$	
BC1	16	9900	10,000 USD	
BC2	32	11,000	10,500 USD	
Links for AALLA Model				
Links	$\mathrm{Omega}\_l\;(\omega^{l})$	$\mathrm{Phi}\_l\;(\phi^{l})$		
L1	10,000,000	0.28 USD		
L2	200,000,000	0.34 USD		
L3	3,000,000,000	6 USD		
Switches for AALLA Model				
Switches	$\mathrm{Sigma}\_s\;(\sigma^{s})$	$\mathrm{Kappa}\_s\;(K^{S})$		
S1	100	0.10 USD		
S2	200	0.15 USD		
S3	300	0.20 USD		
S4	400	0.30 USD		
S5	500	0.40 USD		
S6	600	0.50 USD		

**Table 2:** AMPL and CPLEX solutions for AALLA model in SDN.

(a) Input Dataset # 1 Results Using AMPL and CPLEX Solver								
Switch	Link	Controller	Cluster (Backup) Controller	Switch Assigned to Cluster Controller	Data Size (p)	Cost (USD)	CPLEX Iterations	DDoS Attack Location
S1	L1	C1	BC1	S3	2100 p	45,509	1398	no attack
S2	L2	C2	BC2					S3
S3	L3	C3						no attack
S4								no attack
S5								no attack
S6								no attack
**(b) Input Dataset # 2 Results Using AMPL & CPLEX Solver**								
**Switch**	**Link**	**Controller**	**Cluster** **(Backup) Controller**	**Switch Assigned to Cluster Controller**	**Data size (p)**	**Cost (USD)**	**CPLEX** **Iterations**	**DDoS** **Attack** **Location**
S1	L1	C1	BC1	S3	2100 p	45,509	2986	no attack
S2	L2	C2	BC2	S6				S3
S3	L3	C3						no attack
S4								no attack
S5								no attack
S6								S6
